# Inverse association of long-acting natriuretic peptide with metabolic syndrome in congestive heart failure patients

**DOI:** 10.1186/1758-5996-5-19

**Published:** 2013-04-08

**Authors:** Ji-Hung Wang, Chung-Jen Lee, Jen-Che Hsieh, Yu-Chih Chen, Bang-Gee Hsu

**Affiliations:** 1 School of Medicine, Tzu Chi University, Hualien, Taiwan; 2 Division of Cardiology, Buddhist Tzu Chi General Hospital, Hualien, Taiwan; 3 Department of Nursing, Tzu Chi College of Technology, Hualien, Taiwan; 4 Division of Nephrology, Buddhist Tzu Chi General Hospital, No. 707, Section 3, Chung-Yang Raod, Hualien, Taiwan

**Keywords:** Long-acting natriuretic peptide, Congestive heart failure, Metabolic syndrome, Homeostasis model assessment of insulin resistance

## Abstract

**Aims:**

Long-acting natriuretic peptide (LANP) is one of the peptide hormones in atrial natriuretic peptide (ANP) pro-hormone. Low levels of natriuretic peptide may lead to reduced lipolysis and excessive weight gain in obese patients. The aim of this study was to investigate the relationship between fasting serum LANP level and the metabolic syndrome (MetS) among congestive heart failure (CHF) patients.

**Methods:**

Fasting blood samples were obtained from 186 patients with normal renal function in cardiac clinic outpatients. CHF defined by the American College of Cardiology Foundation and the American Heart Association 2005 Guidelines. MetS and its components were defined using diagnostic criteria from the International Diabetes Federation.

**Results:**

Ninety-eight patients (52.7%) had CHF. There was a tendency of increased fasting LANP levels as the NYHA CHF functional classes increased (*p* = 0.002). Forty-six of the CHF patients (46.9%) had MetS. Fasting LANP level negatively correlated with MetS among CHF patients (*p* < 0.001). Univariate linear regression analysis showed that BUN (*p* = 0.026) positively correlated with fasting serum LANP levels, while body weight (*p* = 0.009), BMI (*p* = 0.004), homeostasis model assessment of insulin resistance (HOMA-IR; *p* = 0.024) and HOMA-β (*p* = 0.001) negatively correlated with fasting serum LANP levels among the CHF patients. Multivariate forward stepwise linear regression analysis of the significant variables showed that the HOMA-β (R^2^ change = 0.292, *p* < 0.001) and HOMA-IR (R^2^ change = 0.081, *p* = 0.019) were independent predictors of fasting serum LANP levels in CHF patients.

**Conclusions:**

LANP level is significantly reduced in CHF patients affected by MetS. HOMA-β and HOMA-IR were independent predictors of serum LANP levels in CHF patients.

## Introduction

There are four peptide hormones derived from the 126-amino acid atrial natriuretic peptides (ANP) pro-hormone: long-acting natriuretic peptide (LANP; N-terminal pro-ANP 1–30), vessel dilator (N-terminal pro-ANP 31–67), kaliuretic peptide (N-terminal pro-ANP 79–98), and ANP (α-ANP) and these peptide hormones have significant diuretic, natriuretic, and blood-pressure-lowering properties in animals and humans [1]. The biologic effects of the four peptide hormones from ANP pro-hormone include vasodilation mediated via enhancing guanylate cyclase activity, with a resultant increase in the intracellular messenger cyclic guanosine monophosphate (cGMP), and inhibit the vasoconstrictive peptide endothelin [2]. ANP also activates cGMP-dependent protein kinase, leading to perilipin and hormone-sensitive lipase phosphorylation, and induces lipolysis [3]. ANP is rapidly cleared from the circulation with a half-life of 3–4 minutes. No receptors for N-terminal pro-ANP are known today; therefore this peptide circulates longer which leads to significant higher concentrations in blood compared to ANP. Thus, circulating levels of N-terminal pro-ANP are less sensitive to the pulsatile secretion of ANP and may better reflect chronic levels of ANP secretion than the rapidly fluctuating levels of ANP [1,2].

The metabolic syndrome (MetS) is associated with increased cardiovascular events [4]. The prevalence of MetS among congestive heart failure (CHF) patients was more than double compared with that in the general population in Japan [5]. A recent study noted that MetS predicted CHF independent of interim myocardial infarction and prevalent diabetes in elderly Finns during a 20-year follow-up [6]. CHF was also associated with insulin resistance independent of diabetes mellitus in a large community-based sample of elderly men [7]. It can be speculated that low levels of natriuretic peptide may lead to reduced lipolysis and excessive weight gain in obese patients, which may be one of the biological alterations that contribute to the development of obesity [8]. Lower plasma N-terminal pro-ANP levels were also associated with the development of insulin resistance (HOMA-IR) and MetS [9]. LANP is one of the N-terminal pro-ANP. There is no study about the association between serum LANP levels and MetS in CHF patients. The aim of this study was to investigate the relationship between fasting serum LANP level and the MetS among CHF patients.

## Material and methods

### Patients

Between October 2009 and December 2009, 186 consecutive clinic outpatients in a medical center in Hualien, eastern Taiwan (80 males and 106 females, age range 37–88 years) were enrolled into this study. The Protection of the Human Subjects Institutional Review Board of Tzu-Chi University and Hospital approved this study. Patients were excluded if they had any acute infection, acute myocardial infarction, pulmonary edema, liver cirrhosis, thyroid disease, chronic renal failure (serum creatinine ≥1.2 mg/dl) at the time of blood sampling or if they refused to provide informed consent for the study.

### Definition of CHF

In the present study, we included patients with stages C/D CHF defined by the American College of Cardiology Foundation and the American Heart Association 2005 Guidelines. Heart failure is a complex clinical syndrome that can result from any structural or functional cardiac disorder that impairs the ability of the ventricle to fill with or eject blood [10]. New York Heart Association (NYHA) CHF functional classes were collected from the medical records. According to the European Society of Cardiology 2007 Guidelines, we further divided them into 2 groups: heart failure with preserved ejection fraction (HFPEF; LV ejection fraction (EF) ≥50%) and heart failure with reduced ejection fraction (HFREF; LVEF <50%) [11].

### Anthropometric analysis

Body weight was measured to the nearest half-kilogram with the patient in light clothing and without shoes. Height was measured to the nearest half centimeter. Waist circumference was measured in a horizontal plane, midway between the inferior margin of the ribs and the superior border of the iliac crest. Body mass index (BMI) was calculated as weight (kilograms) divided by height squared (meters). Bioimpedance measurements of fat mass were performed at the bedside according to the standard, tetrapolar, whole body (hand-foot) technique, using a single-frequency (50-kHz) analyzer (Biodynamic-450, Biodynamics Corporation, Seattle, MA, USA). Measurements were carried out by the same operator; fat mass was collected and analyzed by specific formulas provided by the manufacturer [12-15].

### Biochemical investigations

Fasting blood samples of approximately 0.5 ml for measuring complete blood count (Sysmex K-1000, Bohemia, NY, USA) and other factors were immediately centrifuged at 3000 *g* for 10 min. Serum levels of blood urea nitrogen (BUN), creatinine (Cre), fasting glucose, total cholesterol (TCH), triglyceride (TG), high-density lipoprotein-cholesterol (HDL-cholesterol), low-density lipoprotein-cholesterol (LDL-cholesterol), albumin, globulin, and C-reactive protein (CRP) were measured using an autoanalyzer (COBAS Integra 800, Roche Diagnostics, Basel, Switzerland). Serum LANP (N-terminal pro-ANP 1–30 or pre-pro-ANP 26–55, Phoenix Pharmaceuticals Inc., Burlingame, CA, USA) levels were measured using a commercial available enzyme immunoassay kit (EIA). The limit of detection calculated as the concentration of human LANP corresponding to the blank average minus three standard deviations was 0.1 ng/ml. The inter- and intra-assay coefficients of variation for LANP were 6.2% and 5.4%, respectively [12]. Serum insulin levels were measured using the MEIA (microparticle enzyme immunosobent assay) method by an autoanalyzer (Abbott Laboratories, Abbott Park, IL, USA). Insulin resistance was evaluated using a homeostasis model assessment of insulin resistance (HOMA-IR) as follows: HOMA-IR = fasting plasma glucose (mg/dl) × fasting serum insulin (μU/ml)/405 [15]. HOMA-β (pancreatic β-cell function) was calculated using the following formula: HOMA-β = fasting serum insulin (μU/ml) × 360/fasting plasma glucose (mg/dl) − 63 [16].

### Metabolic syndrome and its components

The prevalence of MetS was defined using the International Diabetes Federation definition [17]. People were classified as having MetS if they had central (abdominal) obesity with a waist circumference ≥90 cm (men) or ≥80 cm (women) (Chinese criteria), and were matching two or more of the following criteria: fasting serum glucose of 110 mg/dl or more, triglycerides of 150 mg/dl or higher, HDL-cholesterol level less than 40 mg/dl in men or less than 50 mg/dl in women, or blood pressure of 130/85 mmHg or higher. The use of antihypertensive medication was considered as high blood pressure in this analysis. Type 2 diabetes was determined according to World Health Organization criteria [18]. A person was regarded as diabetic if the fasting plasma glucose was either 126 mg/dl or more, or if the 2 h glucose during an oral glucose tolerance test was 200 mg/dl or more, or if he/she was using diabetes medication (oral or insulin).

### Statistical analysis

Data are expressed as means ± standard deviation (SD) and were tested for normal distribution by Kolmogorov-Smirnov statistics. Categorical variables were analyzed by the Chi-square test. Comparisons between patients were performed using Student’s independent *t* test (two-tailed) for the normally distributed data or the Mann–Whitney *U* test for the parameters that presented with non-normal distribution (fasting glucose, CRP, LANP). The significance of differences of LANP between groups (NYHA functional CHF classes) was analyzed by the Kruskal-Wallis analysis of variance (AVONA) test. Clinical variables that correlated with serum LANP levels in CHF patients were evaluated by univariate linear regression analyses. Variables that were significantly associated with LANP in CHF patients were tested for independency in multivariate forward stepwise regression analysis. Data were analyzed using SPSS for Windows (version 13.0; SPSS Inc., Chicago, IL, USA). A p-value < 0.05 was considered statistically significant.

## Results

**Figure 1:**
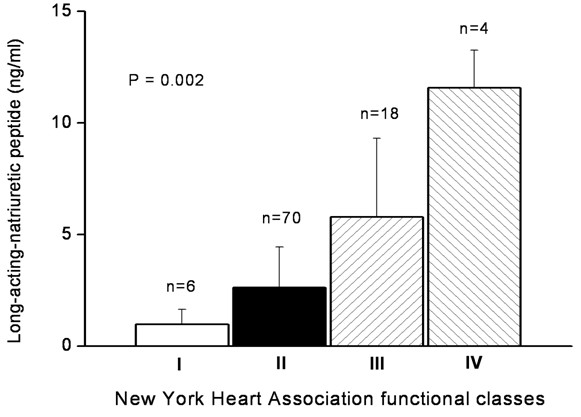
**Fasting serum long-acting natriuretic peptide levels in New York Heart Association functional classes among the 98 congestive heart failure patients.** Data was analyzed by the Kruskal-Wallis analysis of variance (AVONA) test.

The clinical characteristics of the patients with or without CHF are presented in Table 1. Ninety-eight patients (52.7%) had CHF (64 dilated cardiomyopathy, 24 ischemic heart diseases and 10 valvular heart diseases). CHF patients had older age (*p* = 0.037) and higher LANP level (*p* = 0.035) than patients without CHF. There was no difference of the fasting serum LANP levels (*p* = 0.090) between HFREF patients (n = 24; 4.82 ± 3.37 ng/ml) and HFPEF patients (n = 74; 3.03 ± 2.84 ng/ml). However, there was a tendency toward increased fasting LANP levels as the NYHA CHF functional classes increased in CHF patients (*p* = 0.002; Figure 1). The highest value of the fasting LANP level was noted with NYHA CHF functional class IV (11.57 ± 1.68 ng/ml) and the lowest value of the fasting LANP level was noted with NYHA CHF functional class I (0.97 ± 0.61 ng/ml). Although, there is a tendency of lower serum LANP levels in patients with MetS than without MetS according the various CHF classes (I-IV). LANP levels in patients with MetS than without MetS did not differ statistically according the various CHF classes (Table 2).

**Table 1:** Clinical variables of the patients with or without congestive heart failure

**Items**	**No CHF (n = 88)**	**CHF (n = 98)**	***P *value**
Age (years)	62.45 ± 7.75	66.73 ± 11.19	0.037*
Height (cm)	158.35 ± 6.98	160.06 ± 7.73	0.268
Body weight (kg)	71.62 ± 10.48	72.39 ± 13.53	0.761
Waist circumference (cm)	95.11 ± 8.74	96.99 ± 10.25	0.384
Body mass index (BMI; kg/m^2^)	28.57 ± 3.79	28.09 ± 3.85	0.547
Body fat mass (%)	36.19 ± 7.36	36.91 ± 6.89	0.634
White blood count (x1000/ul)	7.01 ± 1.99	6.91 ± 1.75	0.805
Haemoglobulin (g/dl)	13.64 ± 1.82	13.86 ± 1.65	0.549
Albumin (g/dl)	4.50 ± 0.27	4.43 ± 0.24	0.241
Globulin (g/dl)	2.90 ± 0.48	2.94 ± 0.41	0.608
Total cholesterol (mg/dl)	191.66 ± 43.62	192.82 ± 34.03	0.886
Triglyceride (mg/dl)	150.77 ± 84.21	161.10 ± 95.39	0.585
HDL-C (mg/dl)	48.05 ± 13.87	45.53 ± 12.74	0.365
LDL-C (mg/dl)	127.00 ± 43.38	124.94 ± 29.83	0.788
Fasting glucose (mg/dl)	117.73 ± 34.78	123.45 ± 69.92	0.590
Blood urea nitrogen (mg/dl)	16.16 ± 4.58	17.18 ± 4.96	0.310
Creatinine (mg/dL)	0.81 ± 0.20	0.85 ± 0.18	0.202
CRP (mg/dl)	0.28 ± 0.26	0.40 ± 0.59	0.972
Systolic pressure (mmHg)	130.88 ± 7.32	131.35 ± 11.26	0.819
Diastolic pressure (mmHg)	74.49 ± 8.10	75.06 ± 7.60	0.730
Insulin (μU/dl)	11.22 ± 10.85	11.11 ± 7.95	0.462
HOMA-IR	3.08 ± 2.59	3.50 ± 3.68	0.620
HOMA-β	115.55 ± 165.54	94.43 ± 65.74	0.358
LANP (ng/ml)	2.25 ± 2.01	3.77 ± 4.41	0.035*

*P < 0.05 was considered statistically significant after performing the Student *t*-test or Mann–Whitney *U* test (LANP, CRP, fasting glucose, insulin, HOMA-IR, HOMA-β).

CHF, congestive heart failure; HDL-C, high-density lipoprotein-cholesterol; LDL-C, low-density lipoprotein-cholesterol; CRP, C-reactive protein; HOMA-IR, homeostasis model assessment of insulin resistance; HOMA-β, homeostasis model assessment of beta cell function; LANP, long-acting natriuretic peptide.

**Table 2:** Serum long-acting natriuretic peptide levels in the various congestive heart failure classes (I-IV) in patients with and without metabolic syndrome

**Characteristic**		**CHF class I (n = 6)**	**CHF class II (n = 70)**	**CHF class III (n = 18)**	**CHF class IV (n = 4)**
Metabolic syndrome	No	1.14 ± 0.75	3.48 ± 2.19	6.99 ± 2.98	11.57 ± 1.68
	Yes	0.89 ± 0.52	1.90 ± 1.15	1.57 ± 0.90	0
***P *value**		0.812	0.096	0.111	

Data are expressed as means ± standard deviations.

* *P* < 0.05 was considered statistically significant after Mann–Whitney *U* test.

The clinical characteristics and fasting serum LANP levels of the CHF patients are presented in Table 3. Forty-six CHF patients (46.9%) had MetS, whereas the remaining 52 patients (53.1%) did not. CHF patients who had MetS had lower serum fasting LANP levels than those without MetS (*p* < 0.001). LANP levels did not differ statistically by gender distribution, diabetes, hypertension, hyperlipidemia, ARB, ACEI, CCB, β-blocker, thiazide, statin, fibrate, sulfaurea, metformin, aspirin, or clopidogrel drugs used.

**Table 3:** Clinical characteristics and fasting serum long-acting natriuretic peptide levels of 98 congestive heart failure patients

**Characteristic**		**Number (%)**	**LANP (ng/ml)**	***P *value**
Gender	Male	46 (46.9)	3.07 ± 2.59	0.589
	Female	52 (53.1)	4.39 ± 5.53	
Diabetes	No	60 (61.2)	3.32 ± 2.78	0.886
	Yes	38 (38.8)	4.48 ± 6.21	
Hypertension	No	28 (28.6)	5.04 ± 7.04	0.626
	Yes	70 (71.4)	3.26 ± 2.76	
Hyperlipidaemia	No	32 (32.7)	5.18 ± 6.59	0.376
	Yes	66 (67.3)	3.09 ± 2.72	
Metabolic syndrome	No	52 (53.1)	5.51 ± 5.44	<0.001*
	Yes	46 (46.9)	1.80 ± 1.09	
Angiotensin-converting enzyme inhibitor use	No	68 (69.4)	3.58 ± 4.68	0.786
	Yes	30 (30.6)	4.20 ± 3.84	
Angiotensin receptor blocker use	No	62 (63.3)	4.17 ± 5.28	0.820
	Yes	36 (36.7)	3.09 ± 2.22	
β-blocker use	No	46 (46.9)	4.07 ± 5.78	0.548
	Yes	52 (53.1)	3.51 ± 2.79	
Calcium channel blocker use	No	58 (59.2)	4.49 ± 5.35	0.263
	Yes	40 (40.8)	2.73 ± 2.26	
Fibrate use	No	94 (95.9)	3.86 ± 4.48	0.511
	Yes	4 (4.1)	1.66 ± 0.17	
Statin use	No	76 (77.6)	3.82 ± 4.80	0.573
	Yes	22 (22.4)	3.61 ± 2.86	
Thiazide use	No	48 (49.0)	2.96 ± 2.54	0.548
	Yes	50 (51.0)	4.55 ± 5.61	
Sulfaurea use	No	92 (93.9)	3.81 ± 4.54	0.677
	Yes	6 (6.1)	3.23 ± 1.57	
Metformin use	No	82 (83.7)	3.44 ± 2.91	0.935
	Yes	16 (16.3)	5.49 ± 9.01	
Aspirin use	No	56 (57.1)	3.87 ± 5.28	0.585
	Yes	42 (42.9)	3.64 ± 3.01	
Clopidogrel use	No	86 (87.8)	3.80 ± 4.631	0.474
	Yes	12 (12.2)	3.59 ± 2.59	

Data are expressed as means ± standard deviations.

* *P* < 0.05 was considered statistically significant after Mann–Whitney *U* test.

The univariate linear analysis of fasting serum LANP levels in CHF patients is presented in Table 4. BUN (r = 0.317; *p* = 0.026) positively correlated with fasting serum LANP levels, while body weight (r = −0.370; *p* = 0.009), BMI (r = −0.407; *p* = 0.004), HOMA-IR (r = −0.321; *p* = 0.024) and HOMA-β (r = −0.469; *p* = 0.001) negatively correlated with fasting serum LANP levels among the CHF patients.

**Table 4:** Correlation of fasting serum long-acting natriuretic peptide levels and clinical variables by univariate linear regression analyses among the 98 congestive heart failure patients

**Items**	**Beta**	***P *value**
Age (years)	0.214	0.140
Height (cm)	−0.070	0.633
Body weight (kg)	−0.370	0.009*
Waist circumference (cm)	−0.259	0.103
Body mass index (BMI; kg/m^2^)	−0.407	0.004*
Body fat mass (%)	−0.092	0.840
Duration of heart failure (months)	0.050	0.765
White blood count (x1000/ul)	−0.042	0.788
Haemoglobulin (g/dl)	−0.067	0.668
Albumin (g/dl)	−0.100	0.494
Globulin (g/dl)	−0.058	0.692
Total cholesterol (mg/dl)	−0.042	0.776
Triglyceride (mg/dl)	−0.248	0.086
HDL-C (mg/dl)	0.010	0.948
LDL-C (mg/dl)	0.061	0.679
Fasting glucose (mg/dl)	0.218	0.132
Blood urea nitrogen (mg/dl)	0.317	0.026*
Creatinine (mg/dl)	−0.018	0.903
CRP (mg/dl)	−0.162	0.265
HOMA-IR	−0.321	0.024*
HOMA-β	−0.469	0.001*

*P < 0.05 was considered statistically significant after univariate linear analyses.

HDL-C, high-density lipoprotein-cholesterol; LDL-C, low-density lipoprotein-cholesterol; CRP, C-reactive protein; HOMA-IR, homeostasis model assessment of insulin resistance; HOMA-β, homeostasis model assessment of beta cell function.

Multivariate forward stepwise linear regression analysis of the variables that were significantly associated with fasting serum LANP levels among CHF patients showed that HOMA-β (β = −0.486, R^2^ change = 0.292, *p* < 0.001) and HOMA-IR (β = −0.289, R^2^ change = 0.081, *p* = 0.019) were the independent predictors of fasting serum LANP levels (Table 5).

**Table 5:** Multivariate stepwise linear regression analysis of body weight, body mass index, blood urea nitrogen, HOMA-IR and HOMA-β: correlation to fasting serum long-acting natriuretic peptide among 98 congestive heart failure patients

**Items**	**Beta**	**R square**	**R square change**	***P *value**
HOMA-β	−0.486	0.292	0.292	< 0.001*
HOMA-IR	−0.289	0.373	0.081	0.019*

*P <0.05 was considered statistically significant after multivariate stepwise linear regression analyses.

HOMA-IR, homeostasis model assessment of insulin resistance; HOMA-β, homeostasis model assessment of beta cell function.

## Discussion

The results of our study showed that the fasting LANP level was negatively associated with MetS in CHF patients. Pancreatic beta cell function and insulin resistance were independent predictors of the serum LANP level among CHF patients.

Cardiac natriuretic peptides consist of a family of six peptide hormones that are synthesized by three separate genes and then stored as three separate pro-hormones (i.e. 126-amino acid atrial natriuretic peptide (ANP), 108-amino acid B-type natriuretic peptide (BNP) and 103-amino acid C-type natriuretic peptide (CNP) pro-hormones). The ANP pro-hormone contains four peptide hormones: LANP, vessel dilator, kaliuretic peptide and ANP [1]. Cardiac natriuretic peptides are known to play a key role in the regulation of salt and water balance and blood pressure homeostasis [19]. High plasma level of LANP was detected in CHF patients and increased in patients of NYHA CHF functional classes II and III [20]. Our study also noted CHF patients had higher LANP levels and noted a tendency for increasing fasting LANP levels as the NYHA CHF functional classes increased in CHF patients. The highest value of the fasting LANP level was noted in NYHA CHF functional class IV, and the lowest value was noted in NYHA CHF functional class I.

MetS represents a constellation of hypertension, abdominal obesity, impaired fasting glucose, and dyslipidaemia, and it has been shown to be a risk factor for cardiovascular disease [17]. CHF is associated with insulin resistance independent of diabetes mellitus, and the prevalence of MetS is high in CHF patients [5,7,21]. It is conceivable that the increased prevalence of MetS in CHF patients is both the cause and the result of CHF, as activation of both the sympathetic nervous system and rennin-angiotensin system causes the metabolic components [22]. Recent study noted that MetS predicted CHF independent of interim myocardial infarction and prevalent diabetes in elderly Finns, and MetS increases mortality in CHF patients [6,21]. Natriuretic peptides induce natriuresis and diuresis, reduce blood pressure, and, moreover, have powerful lipolytic and lipomobilizing activity in humans [23]. At least two adipocyte lipases are important for controlling lipolysis, hormone-sensitive lipase and adipocyte triglyceride lipase. Perilipin and possibly other proteins of the lipid droplet surface are master regulators of lipolysis, protecting or exposing the triglyceride core of the droplet to lipases [24]. ANP increases the intracellular cyclic guanosine monophosphate (cGMP) concentration, which activates cGMP-dependent protein kinase, leading to perilipin and hormone-sensitive lipase phosphorylation, and induces lipolysis [3,25]. Lower plasma N-terminal proatrial natriuretic peptide levels were associated with the development of MetS [9]. ANP increases the production of adiponectin by human adipocytes, as well as in patients with CHF [26]. Serum adiponectin values were found to correlate inversely with the presence of MetS [14,15]. Our study also noted that CHF patients with MetS had lower fasting serum LANP levels.

Human studies indicated that ANP is removed by the lung, kidney, and liver and degraded by natriuretic peptide receptor-C (NPRC), neprilysin, and insulin-degrading enzyme [27]. The reduced glomerular filtration rate may contribute to raise ANP levels through a reduced renal clearance of the peptides [28]. Our study found that BUN positively correlated with fasting serum LANP levels among the CHF patients. Obese individuals in the cohorts of the Framingham Heart Study were found to have lower plasma N-terminal pro-atrial natriuretic peptide levels than those with normal weight [29]. N-terminal pro-atrial natriuretic peptide was also inversely correlated with BMI in a survey of adult male population from Southern Italy [30]. Our study also found that body weight and BMI negatively correlated with fasting serum LANP levels among CHF patients. ANP increased in pancreatic β-cell death was observed in ANP-treated BRIN-BD11 β-cells [31]. Long-term treatment with ANP inhibited ATP production and insulin secretion in rat pancreatic islets [32]. Lower plasma N-terminal pro-ANP levels were associated with the development of insulin resistance in humans [9]. Our study also showed that HOMA-IR, and HOMA-β negatively correlated with fasting serum LANP levels among CHF patients. The multivariate forward stepwise linear regression analysis of the significant variables showed that HOMA-β and HOMA-IR were the independent predictors of fasting serum LANP levels in our study.

Our study noted low levels of LANP in CHF patients may lead to decreased lipolysis and this may be linked to the development of MetS and noted a tendency for increasing fasting LANP levels as the NYHA CHF functional classes increased in CHF patients. Our study also found that BMI negatively correlated with fasting serum LANP levels among CHF patients. MetS is associated with increased cardiovascular events [4]. Numerous studies have documented an obesity paradox, in which overweight and obese people with CHF have a better prognosis than patients who are not overweight or obese [33-35]. The reasons for the obesity paradox in CHF remain unclear. The complex constellation of physical, metabolic, neuronal, and hormonal alterations in obesity may lead to structural and functional alterations in the heart [33]. There is a need for prospective studies to elucidate mechanisms for this relationship.

Many factors may affect serum LANP levels, such as drugs, clearance of LANP, and associated diseases. Type 2 DM is a heterogeneous disorder with a complex aetiology that develops in response to genetic and environment influences. The pathophysiology of type 2 DM is characterized by impaired insulin secretion, peripheral insulin resistance and excessive hepatic glucose production [18]. Although our study noted LANP level was significantly negatively related to the pancreatic beta cell function and insulin resistance in CHF patients. However, LANP levels did not differ statistically by diabetes in this study. Many factors such as excessive hepatic glucose production, case numbers, and drugs may affect the result. Pharmacological interventions have been shown to influence serum ANP in humans. Beta-receptor antagonists appear to augment plasma ANP and cGMP concentrations [36]. Initiation of atorvastatin in the early phase of an acute myocardial infarction has beneficial effects on decreasing ANP level after 24 weeks treatment [37]. Pioglitazone does not alter the circulating levels of ANP in type II diabetes mellitus patients [38]. Our results did not show a relationship between statins or peroxisome proliferator-activated receptor γ agonists or other drugs (angiotensin receptor blocker, angiotensin-converting enzyme inhibitor, calcium channel blocker, or thiazide diuretic) and serum LANP among CHF patients. Further studies are required to elucidate the relationship between medication and LANP in CHF patients.

Our study has some limitations. Firstly, this study was of cross-sectional design. Therefore, our findings should be investigated in long-term prospective studies before a causal relationship between serum LANP and MetS in CHF patients can be established. Secondly, most studies have focused on ANP effects on lipolysis and adipose tissue. However, lack of LANP effects has been investigated. Thirdly, other N-terminal ANP pro-hormone peptides such as vessel dilator or kaliuretic peptide have the same effects as LANP also needed further investigated. Further studies are needed to show the N-terminal ANP pro-hormone peptides effects on MetS in CHF patients.

## Conclusion

The present study shows there was a tendency of increased fasting LANP levels as the NYHA CHF functional classes increased and a negative association between circulating fasting LANP and MetS among CHF patients. HOMA-β and HOMA-IR were independent predictors of the serum LANP level among the CHF patients. LANP level was significantly negatively related to the pancreatic beta cell function and insulin resistance in these patients.

## Abbreviations

ANP: atrial natriuretic peptide; BUN: blood urea nitrogen; CHF: congestive heart failure; cGMP: cyclic guanosine monophosphate; Cre: creatinine; CRP: C-reactive protein; HDL-C: high-density lipoprotein-cholesterol; HFPEF: heart failure with preserved ejection fraction; HFREF: and heart failure with reduced ejection fraction; HOMA-IR: homeostasis model assessment of insulin resistance; HOMA-β: homeostasis model assessment of beta cell function; LANP: long-acting natriuretic peptide; LDL-C: low-density lipoprotein-cholesterol; MetS: metabolic syndrome; NPRC: natriuretic peptide receptor-C; TCH: total cholesterol; TG: triglyceride.

## Competing interests

All authors declare that they have no conflict of interest.

## Authors’ contributions

Lee CJ researched and analyzed data. Wang JH and Hsu BG designed of the study, interpretation of data and wrote the manuscript. Hsieh JC, Chen YC and Wang JH collected the data. All authors read and approved the final version of the manuscript.
